# Medial Prefrontal Cortex Activation Is Commonly Invoked by Reputation of Self and Romantic Partners

**DOI:** 10.1371/journal.pone.0074958

**Published:** 2013-09-23

**Authors:** Hiroaki Kawamichi, Akihiro T. Sasaki, Masahiro Matsunaga, Kazufumi Yoshihara, Haruka K. Takahashi, Hiroki C. Tanabe, Norihiro Sadato

**Affiliations:** 1 Department of Cerebral Research, National Institute for Physiological Sciences, Okazaki, Aichi, Japan; 2 Department of Physiology, Osaka City University Graduate School of Medicine, Osaka, Osaka, Japan; 3 Pathophysiological and Health Science Team, RIKEN Center for Life Science Technologies, Kobe, Hyogo, Japan; 4 Department of Health and Psychosocial Medicine, Aichi Medical University School of Medicine, Nagakute, Aichi, Japan; 5 Department of Psychosomatic Medicine, Graduate School of Medical Sciences, Kyushu University, Fukuoka, Fukuoka, Japan; 6 Department of Physiological Sciences, The Graduate University for Advanced Studies (Sokendai), Okazaki, Aichi, Japan; 7 Department of Social and Human Environment, Graduate School of Environmental Studies, Nagoya University, Nagoya, Aichi, Japan; Cuban Neuroscience Center, Cuba

## Abstract

The reputation of others influences partner selection in human cooperative behaviors through verbal reputation representation. Although the way in which humans represent the verbal reputations of others is a pivotal issue for social neuroscience, the neural correlates underlying the representation of verbal reputations of others are unclear. Humans primarily depend on self-evaluation when assessing reputation of self. Likewise, humans might primarily depend on self-evaluation of others when representing their reputation. As interaction promotes the formation of more nuanced, individualized impressions of an interaction partner, humans tend to form self-evaluations of persons with whom they are intimate in their daily life. Thus, we hypothesized that the representation of reputation of others is modulated by intimacy due to one’s own evaluation formation of that person. To test this hypothesis, we conducted a functional magnetic resonance imaging experiment with 11 pairs of romantic partners while they viewed an evaluation of a target person (self, partner [intimate other], or stranger [non-intimate other]), made by other evaluators. When compared with strangers, viewing evaluations of self and partner activated overlapping regions in the medial prefrontal cortex. Verbal reputation of self-specific activation was found in the precuneus, which represents self-related processing. The data suggest that midline structures represent reputation of self. In addition, intimacy-modulated activation in the medial prefrontal cortex suggests that the verbal reputation of intimate others is represented similarly to reputation of self. These results suggest that the reputation representation in the medial prefrontal cortex is engaged by verbal reputation of self and intimate others stemming from both own and other evaluators’ judgments.

## Introduction

Reputation plays a key role in human society. Reputation can emerge when information about an actor’s behavior in one of his or her relationships spreads to other partners via an information network [1]. Regarding cooperation behaviors, humans pay attention to others’ reputation for selecting cooperation partners [2], [3]. Humans often rely on the verbal reputation of others for social behaviors related to the evaluation of products [4] or people [5]. As a verbal reputation mechanism is unique to humans, processing of the verbal reputation of others is a pivotal issue in social neuroscience.

Several neuroimaging studies have investigated the neural mechanisms underlying reputation processing. Having a positive reputation, which covaries with high social desirable traits, activates the medial prefrontal cortex (mPFC) and striatum [6]. By contrast, a negative reputation activates the mPFC, anterior insula, and anterior cingulate cortex (ACC) [7]. Given these findings, reputation is commonly represented by mPFC activation [8]. Izuma et al. reported that evaluation of a stranger’s reputation did not activate the mPFC [6]. However, as we select cooperation partners based on their reputation [2], [3], we are able to imagine – and form a representation of – the reputation of others. Thus, it is still unclear how reputation of others invokes reputation representation in the mPFC.

In everyday life, an individual can assess reputation of self by using the responses of others to infer how they think about the individual [9], [10]. This kind of inference is called reflected appraisal [11], [12]. In this sense, reflected appraisal has similar characteristics to reputation representation. Reflected appraisal is constructed using meta-representations of others’ evaluations of a target person [13], and reflected appraisal is primarily dependent on one’s own evaluation [11]. To represent a reputation, humans might use information from both their own evaluations and others’ evaluations, including verbal reputation evaluations made by others. In this type of cognition, one might take an anchoring and adjustment approach, which is defined as adjusting beliefs based on others’ viewpoints from an anchor point based on one’s own beliefs [14]. In this sense, one’s own evaluation, together with others’ evaluations, plays a key role in processing reputation. Previous results showing inactivation of the mPFC when processing the reputation of others vs. processing reputation of self [6] could be due to a lack of one’s own evaluation. Therefore, we expected that one’s own evaluation of the reputation target is utilized to represent the reputation of others. As the motivation for interaction with others promotes the formation of more nuanced, individualized impressions of the interaction target [15], humans tend to form self-evaluations of intimate persons in their daily life. Thus, we hypothesized that activity in the mPFC should increase when reputation information about oneself or an intimate other is being processed, but not when the same information about a stranger is being processed.

To test this hypothesis, we conducted a functional magnetic resonance imaging (fMRI) study with 11 pairs of romantic partners during an adjective judgment task using two 3T MR scanners. Romantic partners were used to gauge the intimacy effect, because romantic love is accompanied by the desire to share emotions and experiences [16], which leads to intimate relationships. Therefore, humans have formed their own detailed evaluation of their romantic partners. In the experiment, each subject viewed photos of three types of evaluation target (self, partner, or stranger), together with an evaluation word ostensibly reflecting the rating of the target. At that time, they were required to judge the social desirability of the rating word irrespective of the target photo.

## Materials and Methods

### Subjects

A total of 11 romantic couples took part in the experiment. The mean ± standard error of the mean (SEM) age was 21.3±0.4 years (22.3±0.7 for males and 20.6±0.5 for females). All subjects had normal or corrected-to-normal visual acuity and were right-handed according to the Edinburgh Handedness Inventory [17] except two female subjects. Subjects were provided with monetary compensation. The study protocol was approved by the ethical committee of the National Institute for Physiological Sciences (Okazaki, Japan). The experiments were undertaken in compliance with national legislation and the Code of Ethical Principles for Medical Research Involving Human Subjects of the World Medical Association (Declaration of Helsinki). All the subjects provided written informed consent. Six subjects were excluded from the reported results (data from 16 subjects are presented here), because one subject reported that they did not believe that the impressions were evaluated by others after the fMRI experiment, four subjects had too much movement (>2 mm) during a run, and the sixth subject had poor task performance (<95% button-press ratio).

### Apparatus for Visual Stimulus Presentation

Visual stimuli were presented using Presentation software 14.4 (Neurobehavioral Systems, Inc., California) implemented on a personal computer (dc7900; Hewlett-Packard Japan, Ltd., Tokyo). A liquid crystal display (LCD) projector (CP-SX12000; Hitachi, Ltd., Tokyo) located outside and behind the scanner projected the stimuli through a waveguide to a translucent screen, which the subjects viewed via a mirror attached to the bed of the MRI scanner. The spatial resolution of the projector was 1,024×768 pixels, with a 60-Hz refresh rate. The distance between the screen and each subject’s eyes was approximately 175 cm, and the visual angle was 13.8° (horizontal)×10.4° (vertical). Responses were collected via an optical button box (HHSC-2×2; Current Designs, Inc., Philadelphia).

### Task Design

The couples participated in two-day sessions, similar to a previous study [6]. The average (±SEM) time between the first and the second experimental days was 10.27±2.22 days. On the first day, the couples took part separately in a self-introduction session, during which they initially completed a self-introduction sheet comprising open-ended questions such as “What do you do in your free time?”, “What is your personality like?”, “What are your goals for the future?”, and “Please pick one problem that modern Japanese society faces and briefly state your opinion for tackling the issue”. After completing the self-introduction sheet, they were required to tell a self-introduction story, which was video recorded. At the beginning of the session, they were told that the information provided would be used by four couples (two groups of four evaluators) to form an impression of the participant. At the first session, we confirmed that the evaluators were strangers to the participants.

On the second day, the subjects took part in the fMRI experiment during which they were presented with the results of the impression evaluations (adjectives) with a photo of the participant (self), his/her partner, or a stranger. The adjectives were presented in a predetermined order. We selected 68 adjectives from 84 items used in a previous study [6]. The adjectives were selected based on the rating results of nine independent evaluators (five males) using a seven-point Likert scale (ranging from 7, highly understandable to 1, not at all understandable). The mean (±SEM) understandability rating of the items selected for the reputation condition was 5.35±0.09. Fifty-two of the 68 items were presented twice, as they were commonly and independently selected by both groups of evaluators. All subjects were told that they would rate the eight evaluators after the fMRI experiment, and that our aim was to investigate the neural mechanisms underlying first impression formation.

In the experiment, we included conditions in which the subjects viewed the same items but were told that the items represented the impressions of four strangers (two male and two female), in addition to themselves and their partner, in order to investigate intimacy effects on neural activation. At the beginning of the fMRI experiments, the subjects were told that the same eight assessors had evaluated these four strangers. The use of four agents in the stranger condition was similar to a previous study [6]. If one agent was used in the stranger condition, the same groups of adjectives would be used for three persons (self, partner, and stranger). This situation might raise the possibility of subjects noticing the experimenters’ manipulation of adjectives. Using four agents in the stranger condition was intended to keep this effect to a minimum. In addition, we confirmed that the subjects did not know the four strangers by interviewing them after the experiments.

To check the validity in terms of impression-formation modulation caused by intimacy, 14 independent evaluators (five females; mean ± SEM age, 21.4±0.6 years [male, 21.1±0.4; female, 22.0±1.5]) rated the degree of impression formation using a visual analog scale (100, well formed; 0, could not form an impression). The average ± SEM ratings were 85.71±2.97, 83.29±4.52, 52.79±4.56, 51.50±5.22, 53.29±5.73, and 51.71±6.04 for self, partner, first stranger, second stranger, third stranger, and fourth stranger, respectively. One-way (target = self/partner/first stranger/second stranger/third stranger/fourth stranger) repeated measures analysis of variance (rmANOVA) showed a significant main effect (*p*<0.05). Post-hoc analysis showed no significant differences between pairs formed with each of the four strangers. However, pairs formed of each of the four strangers and self or partner showed significant differences (between self and first stranger or second stranger, *p*<0.001; partner and fourth stranger, *p*<0.05; and other pairs, *p*<0.01). The impression formations of the four strangers were similar and the impression of self or partner was well formed in comparison.

**Figure 1:**
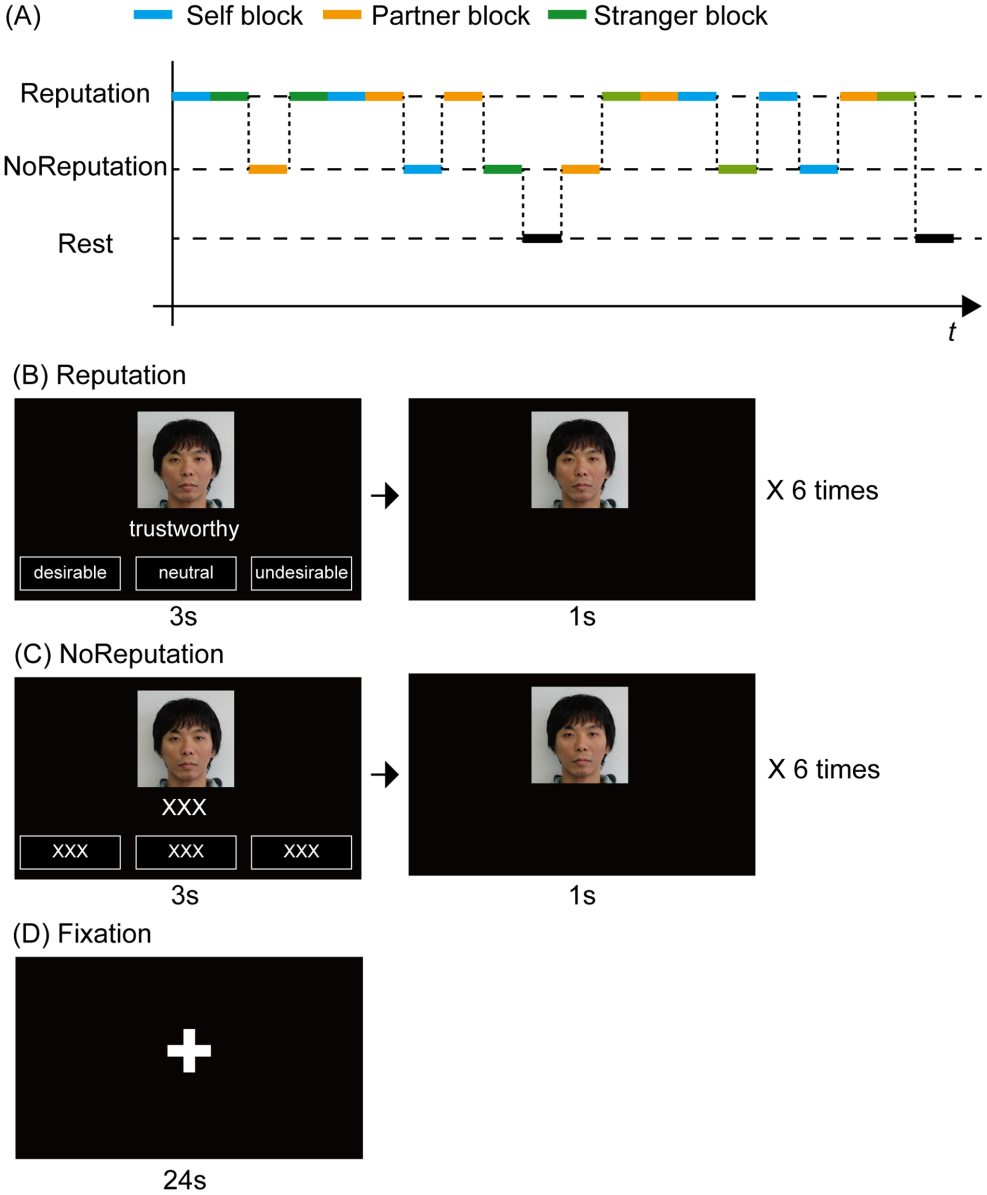
Experimental flow. (A) An example condition sequence is shown. The reputation and no-reputation conditions for three targets (self, partner, and stranger) were presented in a pseudorandom order. (B) Visual stimuli in the reputation condition are shown. A photograph of the evaluation target was presented throughout the reputation condition. The adjectives evaluated by ostensible evaluators and three choices in terms of desirability rating were presented for 3 s in the lower part of the display. Subjects were required to rate the social desirability of the adjectives irrespective of the evaluation target for 3 s. Three seconds after the adjective onset, the adjective and choices disappeared, and the photograph of the evaluation target alone was displayed for 1 s. In the reputation-condition block, six adjectives were displayed sequentially. (C) Visual stimuli in the no-reputation condition are shown. Here, ‘XXX’ was displayed instead of the adjectives and choices in the reputation condition. (D) In the rest-condition block, a fixation cross was presented at the center of the screen for 24 s.

In the fMRI experiments, the subjects were required to evaluate the social desirability of the shown items on a three-point scale (ranging from 3, desirable to 1, undesirable) using the right index, middle, or third fingers during the reputation conditions, irrespective of which person was presented. During the no-reputation condition, the subjects were asked to press a button with their right index finger. Subjects were required to respond within 3 s to each stimulus in the reputation and no-reputation conditions. Four seconds after stimulus onset, the next stimulus was presented. The reputation, no-reputation, and rest blocks were each 24 s in length (Fig. 1).

The reputation conditions were repeated four times for the self, partner, and stranger conditions in each fMRI run. The no-reputation conditions were repeated two times for the self, partner, and stranger conditions in each fMRI run. In addition, a fixation rest block was repeated two times in each run. There were five runs in each session. The participating couples completed the fMRI session simultaneously using hyper-scanning 3T fMRIs positioned side-by-side. The condition sequence for each run was predetermined and counterbalanced across subjects. Two blocks of self, partner, and stranger conditions were never presented in succession within a run. Prior to the fMRI experiment, the subjects took part in a 2-min practice session, in which no photos were included and the adjectives were different from those used in the fMRI experiment. The total duration of the run was 8 min.

### fMRI Data Acquisition

Two 3T scanners (Verio; Siemens, Ltd., Erlangen) were used for the fMRI study. Each subject’s head was immobilized within a 32-element phased-array head coil. fMRI was performed using an echo planar imaging (EPI) gradient-echo sequence (echo time [TE] = 30 ms; repetition time [TR] = 3,000 ms; field of view [FOV] = 192×192 mm^2^; flip angle = 83°; matrix size = 64×64; 39 slices; slice thickness = 3 mm; and total number of volumes = 94). A whole-brain high-resolution, T1-weighted anatomical MR image using magnetization-prepared rapid acquisition gradient echo (MP-RAGE) was also acquired for each subject (TE = 2.97 ms; TR = 1,800 ms; FOV = 256×256 mm^2^; flip angle = 9°; matrix size = 256×256 pixels; and slice thickness = 1 mm).

### fMRI Data Analysis

We used SPM8 revision 4667 (The Wellcome Trust Centre for Neuroimaging; http://www.fil.ion.ucl.ac.uk/spm) implemented in MATLAB 2010a (MathWorks, Inc., Massachusetts) to analyze the functional images. The first four volumes of each fMRI run were discarded because the MRI signal was unsteady. We initially performed motion correction, normalization to the Montreal Neurological Institute (MNI) template, and spatial smoothing (8 mm). After the realignment processes, we checked the head-movement parameters. The task-related activation was evaluated statistically on a voxel-by-voxel basis using a general linear model at the individual level to generate contrast images, which then were incorporated into random-effects analysis at the group level [18].

In the fMRI data analysis, we excluded data from six subjects; thus, data from 16 subjects were analyzed. To investigate the neural correlates underlying reputation representation, we compared the brain activation between the reputation and no-reputation conditions for the self, partner, and stranger conditions. As the mPFC is activated during both positive self reputation [6] and negative self reputation [7], the reputation representation processed in the mPFC should not be influenced by social desirability. As reputation valuation processes were modulated by social desirability [6], we included the average social desirability scores during each reputation-condition block as parametric modulation terms in the individual level analysis for distinguishing reputation representation-related activation from valuation-related activation. The average social desirability score for each block was calculated using the social desirability ratings obtained after the fMRI experiment. After the fMRI experiment, social desirability was evaluated by each subject using a seven-point Likert scale. We then conducted a random-effects analysis using the contrast images produced by the first-level analysis (contrasts of the reputation and no-reputation conditions for the three target types [self, partner, and stranger] and the interaction of the reputation effects).

### Behavioral Data Analysis

We conducted statistical analysis of the social desirability ratings and response times acquired during the fMRI experiment. In this analysis, we performed a one-way (target = self/partner/stranger) rmANOVA of the social desirability scores. We also conducted a two-way (reputation level [reputation/no-reputation]×target [self/partner/stranger]) rmANOVA of the response times for the social desirability judgments made during the fMRI experiment.

## Results

### Rating Results

**Figure 2:**
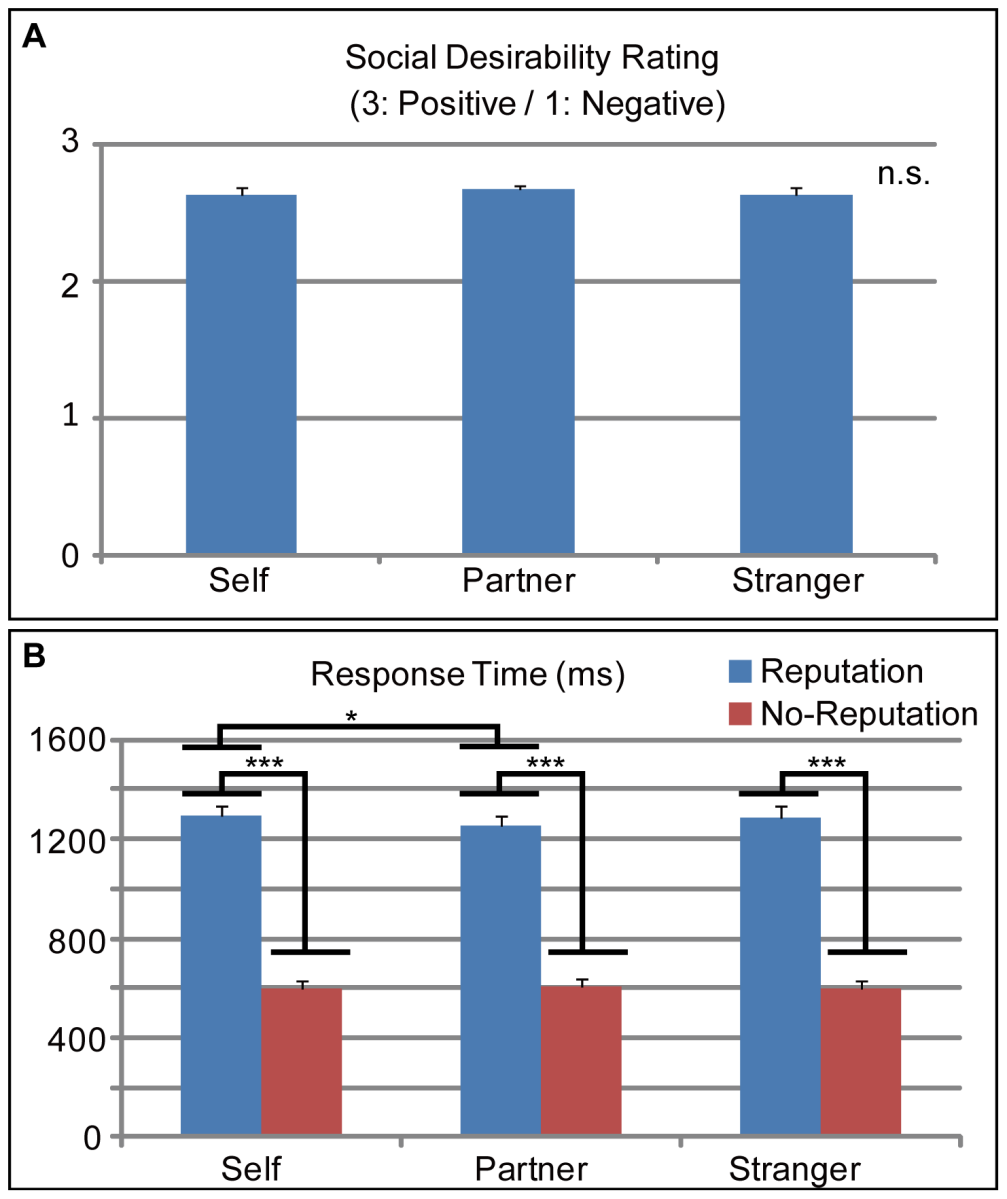
Social desirability judgment data. (A) Average (SEM) values of the social desirability ratings of the reputation words during three conditions. None of the pairs showed significant differences in ratings. (B) Average (SEM) response times for the social desirability ratings (blue bars) and button presses (red bars) during the three conditions. A two-way (reputation [reputation/no-reputation]×target [self/partner/stranger]) repeated measures analysis of variance (rmANOVA) showed significant interaction effects (p<0.05) and a significant main effect of reputation. Post-hoc analysis revealed that each of the three target conditions showed significant differences between the reputation and no-reputation conditions.

During the fMRI experiment, the average (±SEM) social desirability ratings during the self, partner, and stranger conditions were 2.64±0.05, 2.69±0.04, and 2.64±0.04, respectively. The one-way (target = self/partner/stranger) rmANOVA did not show a significant main effect of target (*p = *0.084) (Fig. 2(A)).

Response times for the social desirability ratings for the reputation condition in the self, partner, and stranger conditions were 1290.7±42.6, 1256.2±40.3, and 1288.4±47.0 ms, respectively. For the no-reputation condition, the response times in the self, partner, and stranger conditions were 600.8±29.9, 604.4±31.8, and 598.7±31.1 ms, respectively. A 2 (reputation level = reputation/no-reputation)×3 (target = self/partner/stranger) rmANOVA showed a significant main effect of reputation level (*p*<0.001) and a significant interaction effect (*p*<0.05). There was no significant main effect of target (*p* = 0.415). Post-hoc analyses showed significant differences (*p*<0.001) for all the reputation level pairs in each target, and between the self-reputation and partner-reputation conditions (*p*<0.05) (Fig. 2(B)).

### fMRI Results

**Figure 3:**
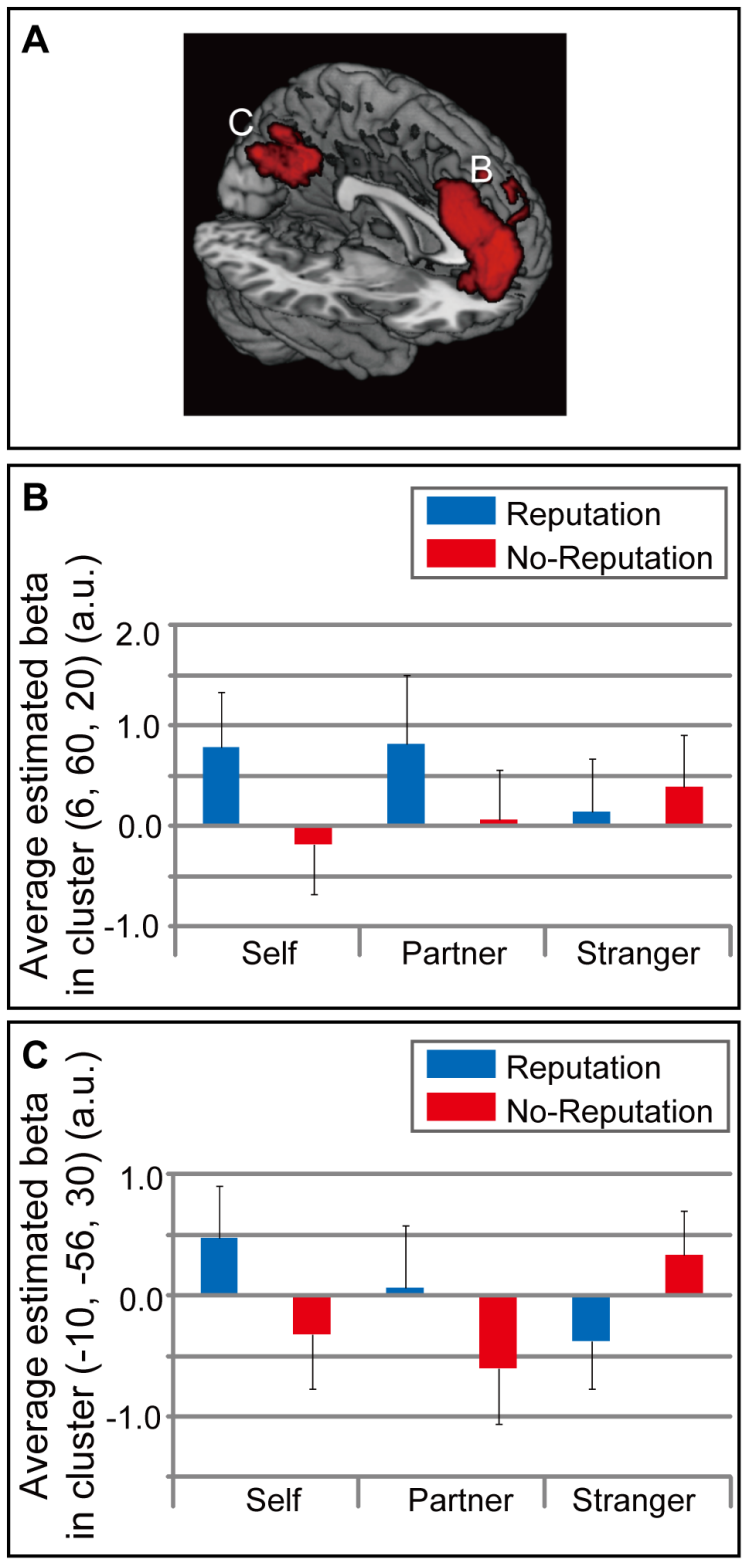
Self reputation-related activation. (A) Significant activation related to the reputation effect during the self condition (self (reputation>no-reputation)) in comparison with the reputation effect during the stranger condition. Activation was located in the medial prefrontal cortex (mPFC: [6, 60, 20]) and precuneus (–10, –56, 30). The statistical threshold was set at an uncorrected *p*<0.01 at the voxel level, and at a family-wise error (FWE) corrected *p<*0.05 at the cluster level. (B) Average (SEM) of the beta values in the cluster located in mPFC is shown. (C) Average (SEM) of the beta values in the cluster located in precuneus is shown.

**Figure 4:**
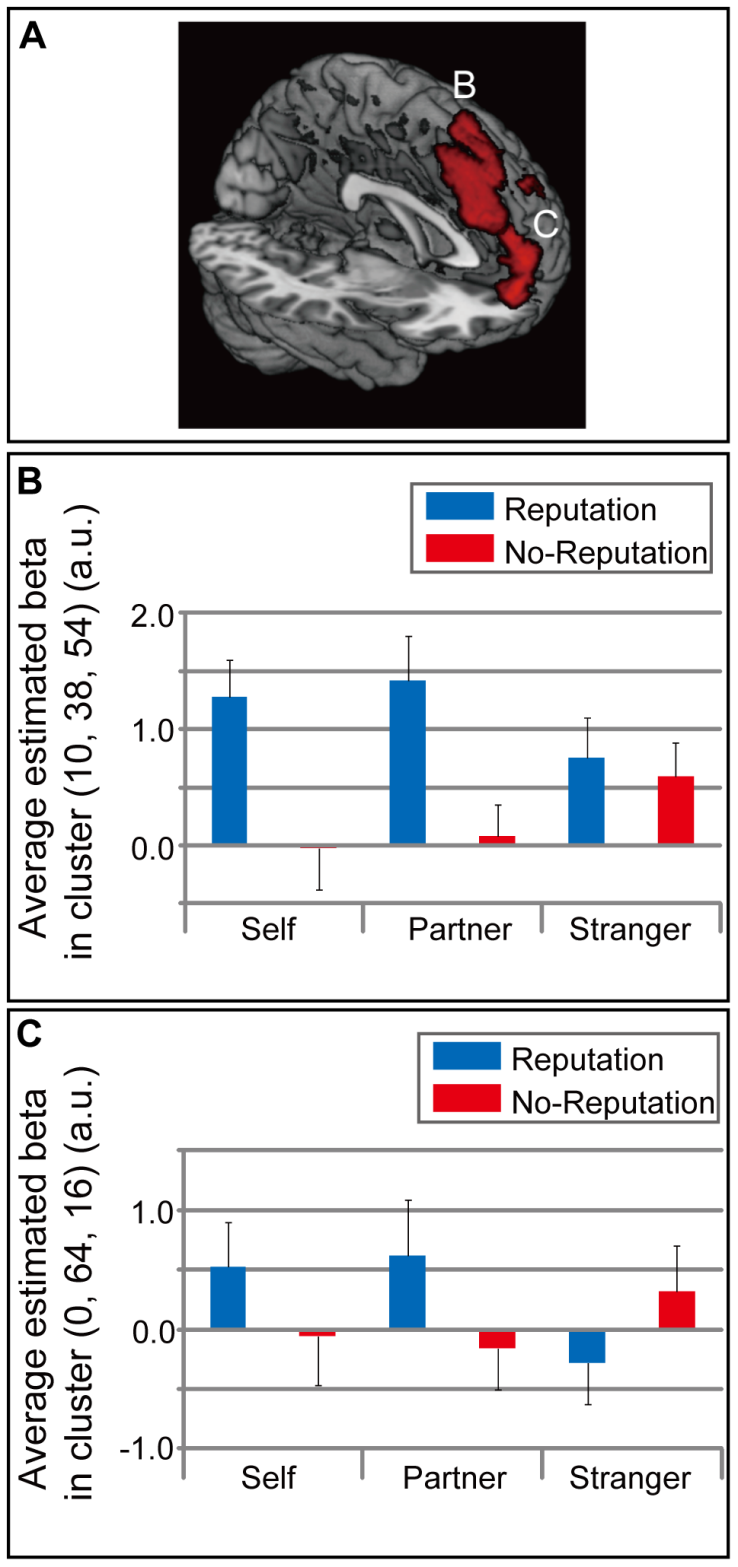
Partner reputation-related activation. (A) Significant activation related to the reputation effect during the partner condition (partner (reputation>no-reputation)) in comparison with the reputation effect during the stranger condition. Two significant clusters were located in the medial prefrontal cortex (mPFC: [10, 38, 54], [0, 64, 16]). The statistical threshold was set at an uncorrected *p*<0.01 at the voxel level, and at a family-wise error (FWE) corrected *p<*0.05 at the cluster level. (B) and (C) show the average (SEM) of the beta values in the two clusters located in mPFC.

**Figure 5:**
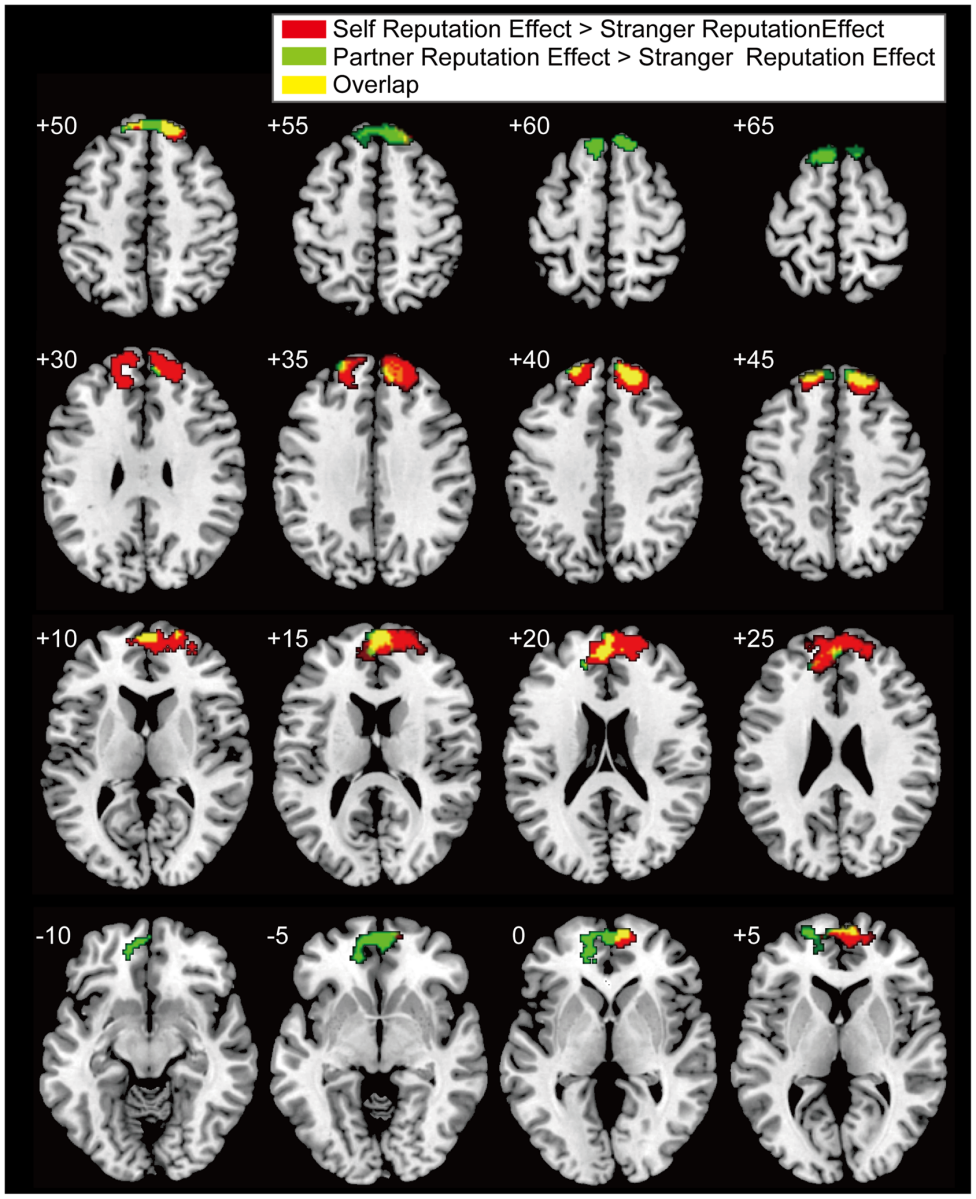
Overlapping activation for the self- and partner-reputation conditions. Significant activation related to the interaction effects of target×reputation in the medial prefrontal cortex (mPFC) is shown. Red and green indicate self-specific (self (reputation – no-reputation)>stranger (reputation – no-reputation)) and partner-specific (partner (reputation – no-reputation)>stranger (reputation – no-reputation)) interaction effects, respectively. Overlapping activation between these two interaction effects is also shown (yellow).

The interaction effects of self vs. stranger and the reputation effect (self (reputation – no-reputation)>stranger (reputation – no-reputation)) showed significant activations in the mPFC and precuneus in the whole-brain analysis (Table 1 and Fig. 3). The mPFC cluster was located in the dorsal part, and mainly occupied the superior frontal gyrus extending to the frontal pole, suborbital sulcus, and cingulate sulcus. The interaction effects of the partner vs. stranger conditions and the reputation effect (partner (reputation – no-reputation)>stranger (reputation – no-reputation)) revealed two significant clusters in the mPFC in the whole-brain analysis (Table 1 and Fig. 4). These two mPFC clusters were located in the dorsal part: the posterior cluster mainly occupied the superior frontal gyrus extending to the cingulate sulcus; the anterior cluster mainly occupied the superior frontal gyrus extending to the frontal pole and suborbital sulcus. The mPFC activation related to the two interaction effects overlapped (Fig. 5). There was no significant activation specific to the stranger-reputation effect, and there was no significant difference between the self- and partner-reputation effects.

**Table 1:** Reputation-related activation.

	Cluster *p* (FWE)	x	y	z	BA	Cluster size	z value
Self (Reputation – No-Reputation)>Stranger (Reputation –No-Reputation)							
*mPFC*	<0.001	6	60	20	BA 10	2621	4.92
*Precuneus*	0.007	–10	–56	30	BA 31	1057	3.84
Partner (Reputation – No-Reputation)>Stranger (Reputation –No-Reputation)							
*mPFC*	0.020	10	38	54	BA 8	891	3.95
*mPFC*	0.041	0	64	16	BA 10	765	3.50

Significant regions of activation revealed by the interaction effects related to the reputation conditions (reputation vs. no-reputation) are shown. The statistical threshold was set at an uncorrected *p*<0.01 at the voxel level, and a family-wise error (FWE) corrected *p<*0.05 at the cluster level. mPFC = medial prefrontal cortex. BA = Brodmann area.

**Figure 6:**
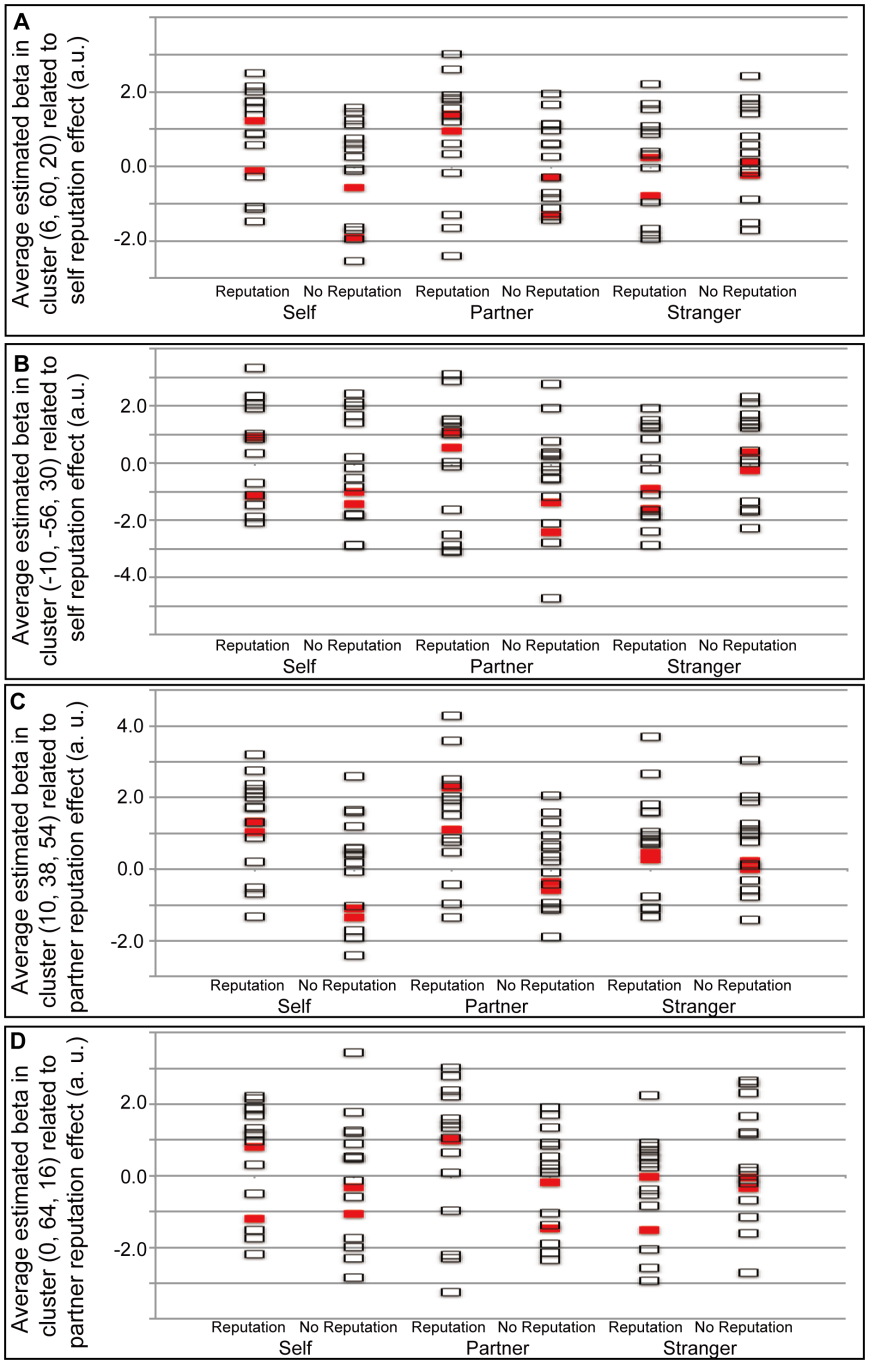
Raw data for average beta values in the four significant clusters. Raw data for average beta values in the mPFC (A) and precuneus (B) related to the self reputation, and the two clusters in the mPFC (C) and (D) related to the partner reputation, are shown. Red and white squares show average beta values in the significant clusters of two left-handed subjects and 14 right-handed subjects, respectively.

Scatter diagrams of the average beta values (Fig. 6) did not reveal any effect of handedness on brain activity in the four significant clusters.

## Discussion

### Behavioral Data: Social Desirability Ratings

As the rating results were not significantly different between the three conditions (self, partner, and stranger), the social desirability ratings were not influenced by the evaluation target. Thus, the subjects properly rated the social desirability of the presented adjectives. Response times for the social desirability ratings showed that response times during the reputation condition were longer in comparison with the no-reputation condition. The longer response times reflected the increased depth of processing required by the social desirability judgment [6]. These results suggest that subjects accurately performed the task during the fMRI experiment.

### Reputation Representation in the mPFC

In the self condition, reputation processing activated the dorsal mPFC in comparison with the stranger condition, consistent with a previous study [6]. This activation was found for the averaged verbal reputation representation of self, and was not modulated by social desirability. Thus, this result supports previously proposed reputation-processing model: the mPFC represents reputation information, which is utilized for valuation processing [8].

The mPFC is a core region for self-referential processing [19], [20], [21]. Task of self-referential thought is a typical type of self-reflection [22]. In self-reflection processes, subjects evaluate whether presented adjectives conform to self. The dorsal mPFC activation was similar to that in previous studies showing the neural correlates underlying self-reflection [19], [20], [21], [22]. This result confirms that self-reflection processing is necessary for reputation representation.

The mPFC is a key node for social cognition [23]. It represents social learning processes and interacts with social information-sensitive areas such as the anterior cingulate cortex (ACC) [24] and superior temporal sulcus (STS) [25], [26]. This kind of learning processes requires theory-of-mind or mentalizing ability [27], [28], [29], [30]. As mPFC activation is related to the mental state as distinct from the physical state [31], the mPFC activation in the present study specifically reflects reasoning while taking into account the perspective of other persons [32] when processing reputation information. In particular, the mPFC represents the accuracy of empathic inference of target emotions [33]; thus, the mPFC activation in the present study might represent perspective-taking of others, which is required for developing a reputation representation based on evaluations made by others.

A previous study showed that reflected appraisal primarily depends on self-evaluation [11]. This was supported by other studies showing that the neural responses underlying reflected appraisal overlapped with those involved in self-evaluation [34], [35]. The mPFC activation in the present study was located near the region where the activation related to reflected appraisal overlapped with the self-evaluation activation in previous studies. Thus, the mPFC activation might include activation related to other’s evaluation in addition to self-evaluation. Filling the gap between others’ and one’s own perspective (i.e., the anchoring and adjustment process) also activates the mPFC [36]. Therefore, as we predicted, the present results showed that mPFC activation when processing one’s own reputation might be related to the comparison between one’s own and others’ evaluations.

### Representation of the Reputation of Intimate Others

In terms of self- and other-reflection, one’s own evaluation of a known other (public image) [37], [38], [39], [40] or intimate other (friend or mother) [34], [41], [42] (other-reflection) in addition to the evaluation of oneself (self-reflection) also activated the mPFC. These results implied that humans form detailed, individualized evaluations of known persons [15] in a way that is similar to self-evaluation. In the present study, compared with strangers, processing of both the verbal reputation of self and the verbal reputation of an intimate other activated the mPFC, which processes reputation representation. This activation pattern is consistent with a previous study, which found common mPFC activation when viewing one’s own face and that of an intimate other [43]. As subjects’ appraisal of others is individualized depending on the motivation to interact [15], in the present study the visual stimuli in the stranger condition were the only targets for which the subjects did not form their own evaluation. The common activation in the mPFC during the reputation representation of both oneself and a romantic partner supports our hypothesis that one’s own evaluation of a target, which is already formed for intimate others, is necessary for developing a reputation representation for others.

Two clusters in the dorsal mPFC (anterior [BA 10] and posterior [BA 8]) were activated by the reputation effect of partner. The anterior dorsal mPFC provides person perception and mentalizing functions, whereas the posterior dorsal mPFC provides attention-related functions [20]. As reputation representation requires knowledge of how others represent the target person, the anterior dorsal mPFC (with person perception and mentalizing functions) might play an essential role, and could possibly interact with attention-related functions represented in posterior dorsal mPFC.

The overlapping activation in the mPFC for reputations of self and partner was shown by the interaction effects of reputation by target vs. stranger. These overlapping activations were located near the area that processes anchoring and adjustment [36]. Similarity between the perceiver and target, one of the characteristics of social closeness, enhances the perceiver’s social projection for the target person and weakens stereotype processing [44] through anchoring and adjustment [36]. Reputation representation processes involving anchoring and adjustment require evaluation related to self- and other-referential processing. Overlapping activation was located in the dorsal part of the mPFC, which is responsible for evaluation in self- and other-referential processing [22]. The present overlapping activation for the reputation of a romantic partner and self indicates that the mPFC processes the reputation of an intimate other by using one’s own evaluation of that person and others’ evaluations of him/her through anchoring and adjustment similar to reputation processing regarding self.

The mPFC is also known to provide functions for other target person-specific processes, such as trust [25], [26]. mPFC activation fluctuates with the amount of trust that one should is statistically predicted to have in others [25], [26]. In the present study, the reputation and no-reputation conditions presented images of the same target persons. This might evoke similar trust feelings in subjects. The contrast between the reputation and no-reputation conditions was intended to cancel out such target person-specific processing. This contrast was expected to provide only reputation effects caused by subjects’ belief in the adjective being considered by other evaluators. Furthermore, the overlapping activation in the mPFC between the partner and self conditions shows intimacy modulation for reputation representation.

### Self-specific Activation

The precuneus was activated specifically during self-reputation processing. The precuneus serves a wide range of functions, including episodic memory retrieval and self-processing operations [45]. As episodic memory is used for the storage and recall of previously experienced events, it has autobiographical reference [46] (i.e., it requires retrieving and constructing information related to the self). Precuneus activity during episodic memory retrieval [47] might reflect such self-related processing. In addition, imagining the future, which also requires elaborating upon information related to oneself, showed overlapping activation with episodic memory retrieval in the precuneus [48]. In terms of self-processing operations, self-referential judgment of adjectives (self-reflection) also activates the precuneus [49]. In addition to these task-dependent activations, the precuneus shows prominent activation during resting states (task-independent activation) [50]. This baseline activation in the precuneus during resting states (default mode-network activation) is associated with the representation of the world around us [50]. The default-mode network includes the mPFC, which was activated during the self and partner reputation conditions in the present study, in addition to the precuneus [50]. These two areas, the mPFC and precuneus, are linked according to diffusion tensor image (DTI) fiber tractography and functional connectivity [51]. In terms of comparisons between self-reflection processes and the default-mode network, the mPFC shows greater activity for the former, whereas the precuneus extending to the posterior cingulate cortex shows greater activity for the latter and shared activation for self reflection and the default-mode network [52]. This differential activity pattern suggests that the precuneus activity for reputation of self might reflect default network-related activation. Taken together, these findings suggest that the precuneus might play a key role in the mental representation of the self. Thus, in the present study, the precuneus activation that was specific to the self-reputation condition could represent the formation of the mental representation of the self caused by reputation of self.

### Conclusion

As predicted, the present results showed that the neural correlates underlying verbal reputation representation of others are modulated by intimacy. This intimacy effect suggests that we represent the reputation of intimate others made by third parties in a way that is similar to how we process reputation of self. Due to the intimate relationship between romantic partners, we develop our own evaluation of intimate others. These results suggest that our own evaluation, together with others’ evaluations, is necessary to represent the reputation of others.
